# Integration of ear and hearing care services in low- and middle-income health systems: a systematic review and qualitative synthesis

**DOI:** 10.1080/16549716.2026.2633877

**Published:** 2026-03-06

**Authors:** Carmen de Kock, Lucy Gilson

**Affiliations:** a Department of Pathology, Division of Human Genetics, University of Cape Town, Cape Town, South Africa; b School of Public Health, Health Policy and Systems Division, University of Cape Town, Cape Town, South Africa

**Keywords:** Ear and hearing care integration, health system strengthening, barriers, enablers, low-resource settings

## Abstract

Hearing loss is a global public health burden and mostly affects those living in low- and middle-income countries (LMICs). One approach to address ongoing challenges is the World Health Organization’s recommendation for the integration of ear and hearing care (EHC) services into healthcare packages. However, little is known about EHC integration approaches, particularly in LMICs additionally, these approaches have not been investigated through a health systems lens. This qualitative review aimed to describe the various approaches to the EHC service integration in LMICs and to identify enabling and constraining factors. We reviewed 17 studies, with a focus on LMICs, using adaptations of the Valentijn integration and World Health Organization EHC frameworks, following the PRISMA guidelines. Our investigation showed that most integration approaches were at micro or individual level. Enabling factors for integration of EHC services were training, mentorship, collaboration, technology, inclusion of EHC in healthcare packages and investment in EHC services. Barriers were challenges with training, facilities and equipment, policy implementation and resourcing of EHC services. We further described factors influencing healthcare seeking behaviour and the use of integrated EHC services, such as access and ability to pay, referral systems and communication and awareness. This study describes the complex nature of EHC integration and ways to support integration. Key considerations are the level of integration, training to address workforce issues and factors influencing service utilisation as we work towards health system strengthening.

## Paper Context

- **Main findings:** This review explored experiences with ear and hearing care service integration into the wider health system in low-resource settings and highlighted the importance of addressing integration at all levels of healthcare including micro, meso and macro levels. Comprehensive and appropriate training programmes for community health workers can address staff shortages and support integration at micro-level.
- **Added knowledge:** The inaccessibility of ear and hearing care services impacts the usage of services. Furthermore, issues that can potentially impact healthcare seeking behaviour should be identified and addressed within the integration process.
- **Global health impact for policy and action:** The global recommendation for early detection and treatment of hearing loss, such as newborn hearing screening programmes, can only be effective if adapted as national policy and incorporated into primary healthcare packages. The lessons learnt, such as exploring appropriate mentorship programmes to support integration can aid policymakers in developing and implementing policies, which strengthen ear and hearing care services through appropriate integration approaches.

## Background

Hearing loss (HL) affects approximately 1.5 billion people with a predicted 1.5-fold increase in the next few decades due to an ageing global population, noise-induced and ototoxic hearing loss, common ear infections and other causes [1]. Yet, many causes of HL are preventable, especially in children younger than 15 years, through public health measures, such as immunisation, adequate referral systems, health promotion and education [2–6].

Approximately 80% of people with moderate to higher levels of HL live in low- and middle-income countries (LMICs), adding pressure to healthcare systems that are already severely under resourced [1,6,7]. EHC services in LMICs are fragmented, often provided by limited specialised healthcare workers and not incorporated into national healthcare packages [1]. A recent study which evaluated the ear and hearing care (EHC) workforce in 141 countries over the past 10 years, showed that most LMICs have access to less than one audiologist per million people compared to more than 10 audiologists per million people in most high-income countries (HICs) [8]. Integrating EHC services into healthcare packages at primary level complemented with inclusion at all levels could improve access, equity, quality of care, acceptability of services and efficiency [1,9–17]. Service integration becomes relevant to, and necessary for, health system strengthening when it has a long-term impact on health services, leading to a more improved and responsive health system [10]. The nature, level and approach to integration of services varies across healthcare interventions and settings [9,18]. However, such integration can be understood as entailing managerial or operational changes to health systems that seek to bring together stakeholders or processes through inputs, organisational arrangements, management at operational level and the delivery or organisation of service functions towards improving efficiency and quality of care to achieve health system goals [15,17]. The World Health Organization (WHO) has highlighted HL as a global public health priority and recommends the integration of EHC into universal health coverage; however, this is challenging due to the lack of national policies, limited awareness, poor access to treatment and inadequate supply of healthcare professionals [1,6,19]. To our knowledge, this is the first in-depth systematic review of the experiences of EHC integration broadly defining EHC services as health promotion, prevention, identification, management and rehabilitation extending to education, communication and support structures for those affected by HL [1]. Most studies have focussed on issues, such as health service delivery, the effect of audiological treatment on quality of life and social impact, technology and telehealth applications, or early intervention approaches [19–27]. This review purposefully adopts a systemic lens, recognising the health system as a complex, dynamic system comprising interconnected building blocks [11,13] and interconnected hardware and software [28].

### Aim

To conduct a systematic review and qualitative synthesis of literature on the integration of EHC into health systems by addressing the question, ‘What approaches to integration of ear and hearing services have been implemented in LMICs and what factors have influenced these experiences?’.

### Objectives

1. To describe the approaches to the integration of ear and hearing healthcare services.
2. To determine the factors influencing the integration of EHC services into health systems.
3. To identify the lessons that can be learnt for future EHC integration efforts.

## Methods

We used a qualitative systematic review approach. Such an approach addresses broad questions and aids in guiding evidence-based decision-making using proven scientific methodologies and allowing for comparison across countries, contexts and time to highlight patterns which can be considered for relevance and generalisability [29–31]. This review used the Preferred Reporting Items for Systematic Reviews and Meta Analyses (PRISMA) guidelines [32].

### Article identification

The databases searched included PubMed, Scopus, Web of Science, Cochrane Library and Google Scholar supplemented by citation searching. Search terms or keywords were generated from the research question using Medical Subject Headings (MeSH) terms, such as public health or delivery of healthcare, integrated, systems integration, health services research, ear and hearing care services or audiological services, developing countries or lower income countries including LMIC names according to the World Bank combined with Boolean search strategies (search strategy in Supplementary 1) [33]. The literature search was initially conducted from July to August 2022 and updated in January 2024. The identified articles were exported to Endnote (v.20.4).

### Article selection

The eligibility criteria are depicted in Table 1. The systematic review process followed methods previously described by Moher [34]. These steps entailed database searching by CDK using predetermined key terms, removing duplicates and screening the abstracts and text of remaining articles for their relevance [34]. Eligible articles underwent a full-text review (CDK) with discrepancies resolved by discussions (CDK and LG) and those that did not meet the inclusion criteria were excluded from the study. Given concerns about what criteria to use for the appraisal of qualitative literature [35], we opted not to implement a quality checklist but only included published peer-reviewed articles.

**Table 1.** Inclusion and exclusion criteria.

Inclusion criteria	Exclusion criteria
Articles available in English language.	Not in English
Open-access journals or journals accessible through the University of Cape Town.	Not retrievable
Articles from low-and-middle-income countries or those with a focus on low- and middle-income countries.	In high-income country
Studies from 2017, when the WHA passed a resolution on the prevention of deafness and hearing loss, until 2024.	Published before 2017
All study design types were included.	Articles that did not undergo a peer-review process
	Article did not describe ear and hearing care and integration

### Data extraction, analyses and synthesis

Conceptual frameworks have been shown to be valuable in aiding health system researchers to frame and extract relevant information from empirical research [36]. We explored integration using two relevant frameworks to support a comprehensive overview of the EHC integration:

1. The Valentijn integration framework describes the dimensions and level of integration while considering the relationships among stakeholders [16].
2. The WHO framework for EHC, which builds on the original WHO Health System building blocks framework, was used to assist in identifying system factors influencing integration [1].

The data extraction form, based on the frameworks, supported thematic analysis of the approaches to integration and barriers/enablers. However, we also applied an inductive thematic analysis approach, driven by the data [36], to develop subcodes within the main themes, as well as to identify additional factors influencing the experiences reported. Given the review approach, key findings can potentially be broadly generalisable across country settings [37].

### Defining integration approaches

**Figure 1.**
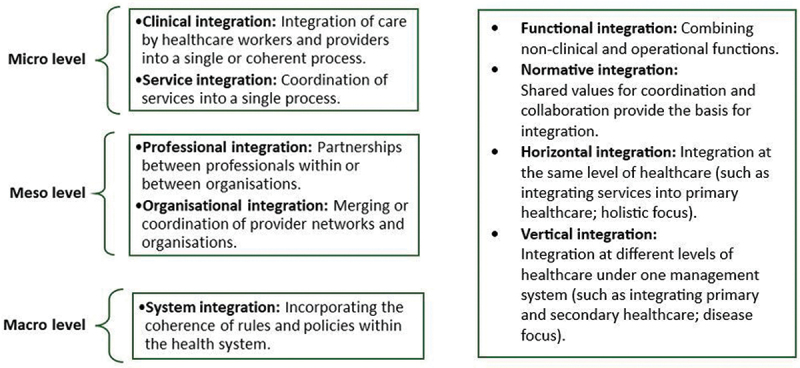
Summary of integration approaches [9,16,17,38].

We applied various concepts of integration to better understand the experiences examined, recognising complexities in the nature, level, and approaches to integration. Integration can, first, be considered at three levels namely micro, meso and macro [16]. At micro-level, the aim is to support the provision of care to individuals; at meso-level, to offer care for specific populations who have the same disease or conditions; and at macro-level, to offer care for entire populations [9]. Figure 1 summarises the approaches to integration that may be used within and across these levels.

## Results

### Search results

**Figure 2.**
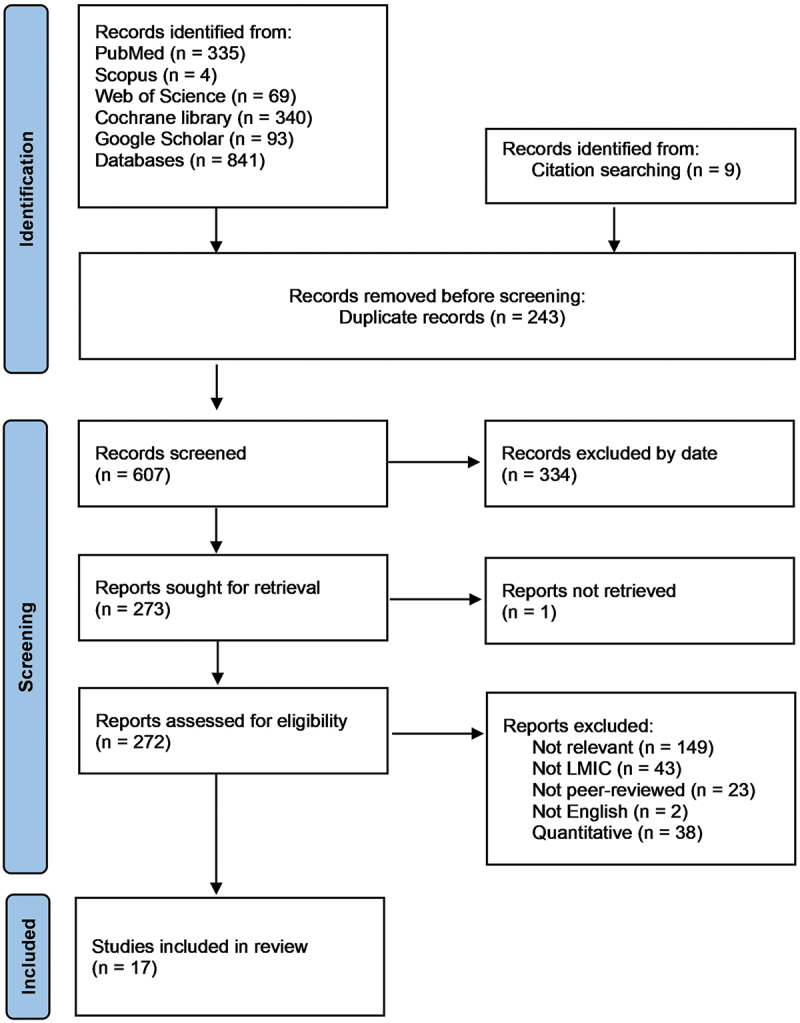
PRISMA diagram illustrating the searching strategy. LMIC: low- and middle-income country.

Database searches yielded 850 articles in total, 243 duplicates were removed, 334 articles were excluded by date, and one article was not retrievable. After full-text review of 272 articles, only 17 articles met the inclusion criteria (PRISMA flowchart, Figure 2).

### Description of articles

The studies described integration of EHC services in rural or low-resource settings in countries, such as India, Peru, South Africa, Malawi, Nigeria, Ghana, Uganda and Cambodia. Five studies were scoping reviews, four qualitative studies, four quantitative studies, three mixed-method studies, and one cross-sectional descriptive study (Table 2).

**Table 2.** Summary of included articles.

	Study details					Integration approach			Enablers and barriers of integration		
No	Title	Author, year	Type of study	Country	Aim	Concept of integration	Description of intervention	Target population	Enablers	Barriers	Healthcare seeking behaviour
1	The role of community health workers in addressing the global burden of ear disease and hearing loss: a systematic scoping review of the literature.	O’Donovan, J. et al. (2019)	Scoping review	Multi-country including LMICs	To identify the role CHWs in the improvement of access to EHC services in LMICs and underserved areas.	Integration of CHWs into ear and hearing care services	Task shifting to cadres of HCWs with less specialised training (CHWs)	CHWs in low-resource settings	Training supports integration.	EHC not prioritised in policies. Lack of HCWs. Inadequate training and support.	Ability to pay. Community participation and awareness.
2	Tele Otology in India: Last 10 Years-A Scoping Review	Angral, S. et al. (2021)	Scoping review	India	Evaluate tele otology and tele audiology to provide EHC services in India.	Integration of tele audiology in EHC services in low resource settings.	Tele audiology interventions using technology for remote EHC service delivery.	Low-resource settings	Training. Collaboration improved acceptability and implementation. Technology improves screening, diagnosis and referrals. Travel to clinics.	Lack of HCWs. Challenges with training. Lack of equipment and infrastructure. Lack of funding	Ability to pay.
3	Impact of COVID-19 pandemic on audiology practice: A scoping review	Aggarwal, K et al. (2022)	Scoping review	Multi-country including LMICs	Explore the impact of COVID-19 pandemic on audiological services and identify challenges.	To assess the influence of the COVID-19 pandemic on audiological practices and identify the unique challenges experienced.	No intervention	Audiologists	_	Lack of training.	_
4	Barriers to access to ear and hearing care services in low- and middle- income countries: A scoping review	Waterworth, C. J. et al. (2022)	Scoping review	LMICs	To identify barriers to accessing EHC services and identify potential solutions.	_	Programmes that support EHC services	People affected by hearing loss and caregivers; EHC HCWs.	Supervision adds to staff retention, competency, motivation and satisfaction. Allocate sufficient budget for EHC services.	Lack of HCWs. Lack of training in communication and counselling.	Access and ability to pay. Lack of knowledge and awareness. Limited referral attendance.
5	Community-based (CB) assessment and rehabilitation of hearing loss: A scoping review	Eubank, T. N. et al. (2022)	Scoping review	Multi-country (LMICs)	To describe community-based hearing rehabilitation.	To describe the implementation of community-based hearing care.	To describe the implementation of community-based hearing care.	Resource-limited communities	Training. Feedback system. Collaboration between CHWs and HCWs.	_	_
6	Audiological follow-up in a risk-based newborn hearing screening programme: An exploratory study of the influencing factors	Kanji, A. and Krabbenhoft, K. (2018)	Qualitative	South Africa	To investigate the positive and negative factors which influence caregivers to return for follow-up of high-risk infants in a risk-based new-born hearing screening programme.	To explore the reasons for follow-up of high-risk infants and challenges experienced by caregivers to attend follow-up visits relating to risk-based hearing screening.	Risk-based hearing screening	Caregivers of infants attending follow-up visits.	_	_	Access and ability to pay.
7	Providing the Best Audiological Care and Creating Sustainability in Peru	Holst, J. E. et al. (2020)	Qualitative	Peru	To describe the needs of the community from the perspective of the HWCs and identify plans for sustainable EHC.	Coordination of HWCs to implement programmes in rural communities.	NPO Idaho Condor Humanitarian who works in communities to identify health needs.	Rural communities	Inclusion of EHC in healthcare package. Professional collaboration. Approaches to EHC service delivery consider local context.	_	Communication and awareness.
8	Barriers and facilitators influencing hearing help-seeking behaviours for adults in a peri-urban community in South Africa: a preventive audiology study	Mtimkulu, T.K. et al. (2023)	Descriptivequalitative	South Africa	To understand the barriers and facilitators contributing to healthcare seeking.	_	No intervention	Hearing impaired patients	_	_	Access and ability to pay. Community-based services built trust.
9	Access to ear and hearing care services in Cambodia: a qualitative enquiry into experiences of key informants	Waterworth, C. J. et al. (2024)	Qualitative	Cambodia	To understand the availability and uptake of EHC services in Cambodia.	_	No intervention	EHC service providers	Public-private partnerships for training.	_	Access and ability to pay. Proper referral mechanisms.
10	Knowledge and cultural beliefs of mothers regarding the risk factors of infant hearing loss and awareness of audiology services	Govender, S. M. and Khan, N. B. (2017)	Quantitative	South Africa	To describe the knowledge of mothers pertaining to the risk factors associated with hearing loss, their awareness of EHC services and the influence of cultural beliefs.	Public awareness	No intervention	Mothers of infants	_	Challenges with policy implementation.	Limited knowledge. Awareness of EHC services.
11	Identifying hearing impairment and the associated impact on the quality of life among the elderly residing in retirement homes in Pretoria, South Africa	Govender, S. M. and De Jongh, M. (2021)	Quantitative	South Africa	To determine the presence of hearing impairment in elderly homes and its impact on quality of life.	Integrating hearing screening for the elderly.	Hearing screening and fitting hearing aids.	Vulnerable populations (elderly)	_	_	_
12	Reasons for low uptake of referrals to ear and hearing services for children in Malawi	Bright, T. et al. (2017)	Mixed methods	Malawi	To explore the uptake of referrals to EHC services among children and barriers for lack of referral uptake.	Integration of CHWs to conduct EHC screening for further referrals.	Task shifting through training CHWs to assist with referrals	Thyolo district in Malawi	Training of CHWs.	Too few staff. Limited infrastructure, equipment and support.	Access to facilities. Effective referrals. Lack of information and awareness. High OOP.
13	Childhood hearing loss; a need for primary healthcare	Ogunkeyede, S. A. et al. (2017)	Cross-sectionaldescriptive study	Nigeria	To describe the experiences of parents/caregivers in accessing EHC services.	Accessibility of EHC services by parents/caregivers of children with hearing impairment.	No intervention	Person-focused. Parents/caregivers of hearing-impaired children.	Collaboration	Limited EHC staff.	Lack of awareness of EHC services and knowledge of hearing loss. Cost of EHC. Including CHWs in referral systems. Inaccessibility of EHC services.
14	Enhancing Ear and Hearing Health Access for Children with Technology and Connectivity	Swanepoel, W. (2017)	Quantitative	South Africa	To assess improved access to EHC through community-based screening and technology.	Community-based screening and technology can improve access to EHC in underserved communities.	mHealth	Communities	Technology improves training, quality of care and access.	Lack of equipment, infrastructure, staff.	_
15	Interregional Newborn Hearing Screening via Telehealth in Ghana	Ameyaw, G. A. et al. (2019)	Quantitative	Ghana	To assess the feasibility of using telehealth to expand EHC services.	Implementing telehealth to improve access to EHC.	Comparing the use of telehealth to conventional methods to screen for hearing loss.	Healthcare facilities delivering NBHS.	Training and support. Collaborative centres. Inclusion of NBHS in healthcare packages.	Lack of HCWs.	_
16	Training, supervision and performance of Community Health Workers in the delivery of ear and hearing care to 321 community members in rural Uganda	O’Donovan, J. et al (2021)	Mixed methods	Uganda	Evaluation of training and service delivery of CHWs.	To provide training to CHWs to conduct hearing screening and evaluate the service provided.	CHWs were trained in hearing screening.	CHWs	Training supports task shifting/sharing. Staff support assist with integration. Collaboration at various levels.	Training not adequate.	Including CHWs in referral systems.
17	Clinical attendance rate at a tertiary adult audiological service in South Africa	Mubina Khan, M. et al (2023)	Mixed methods	South Africa	To explore the rate of attendance and factors influencing attendance at an audiology clinic.	_	No intervention	EHC patients	_	_	Access to EHC facilities.

### Approaches to integration of ear and hearing healthcare services

Most papers reported EHC integration experiences at the micro-level, with no experiences reported at the macro-level (Table 3). At the micro level, service integration was the most reported approach to integration, followed by clinical and professional integration.

**Table 3.** Summary of integration approaches.

Approaches to integration	Level of integration	
	Micro-level	Meso-level
Clinical integration	1; 4; 12; 16	
Service integration	1; 2; 6; 11; 14; 15	
Professional integration		5; 7
Normative integration		7
Organisational integration		2; 7
Functional integration		
System integration		

Note: Numbers in table indicate paper reference.

#### Integration at micro-level

At micro-level, four papers presented studies illustrating clinical integration through task shifting or sharing [39–42], a workforce intervention recommended by the WHO to address healthcare worker shortages [1]. Two papers considered integration through the inclusion of community health workers (CHWs). This cadre usually offered a wider range of healthcare services, to which EHC was added after they had received specialised training – in Uganda, to conduct hearing screening [41] and in Malawi, to support referral management [42]. In Uganda, CHWs’ new role in EHC screening meant this service was incorporated into community-based programmes (reflecting a horizontal integration approach) [41]. In Malawi, in contrast, referral management by CHWs (at community level) to tertiary facilities reflected a vertical approach [42]. The studies conducted by Waterworth et al. [40] and O’Donovan et al. [39] also reported clinical integration approaches that entailed integrating CHWs into EHC services, such as screening and treatment of ear disease.

Six papers reported studies illustrating service integration at the micro-level with two studies describing vertical approaches and four studies describing horizontal approaches (Table 3). Furthermore, two of the papers reported interventions to improve access to EHC services: using tele-audiology in India [43] and smartphone technology to conduct hearing screening in South Africa [44]. The latter study showed that the implementation of community-based hearing screening performed by CHWs using advanced technology was as effective as hearing screening conducted by specialised healthcare workers (HCWs) [44]. A second South African study evaluated a vertical integration approach that entailed screening infants at risk of hearing loss and determining the factors, which influenced follow-up attendance at specialised facilities by the high-risk patients [45]. Another study in South Africa considered service-level integration through implementing hearing screening programmes targeting the elderly to evaluate the prevalence of hearing loss in this population and its effect on quality of life [46]. Hearing screening and counselling conducted by trained CHWs was successfully incorporated into community screening services in India [39]. One paper reported an evaluation of the integration of EHC services at community level using tele-audiology by CHWs and audiologists in India, intended to address service delivery challenges in rural communities [43]. Another paper reported an experience of integration, from two regions in Ghana, focussing on newborn hearing screening (NBHS) services and using tele-audiology in hospital settings, compared to conventional [47]. This study demonstrated improved access to EHC for rural populations through integrating tele-audiology.

#### Integration at meso-level

Only three papers examined meso-level integration, mostly focusing on professional approaches to integration (Table 3). In India, organisational integration efforts between schools and HCWs facilitated the successful implementation of remote hearing screening programmes at schools by audiologists [43]. Professional integration in the rural villages of Cusco, Peru, involved a diverse group of HCWs from the medical, dental and audiology fields providing clinical services [48]. These CHWs and HCW teams had shared values (normative integration) and worked towards the common goal of providing quality and sustainable EHC services [48]. The same paper illustrated organisational integration for EHC services, through partnerships between the local Peruvian health professionals and/or government and the nonprofit organisation, Idaho Condor Humanitarian, bringing together the expertise and experience of these partners to support integration [48]. Interprofessional collaboration assisted with improving access to EHC services in India, as CHWs could deliver services to rural areas using tele-audiology with the support of hearing care specialists [49].

### Factors enabling integration of ear and hearing care services

**Figure 3.**
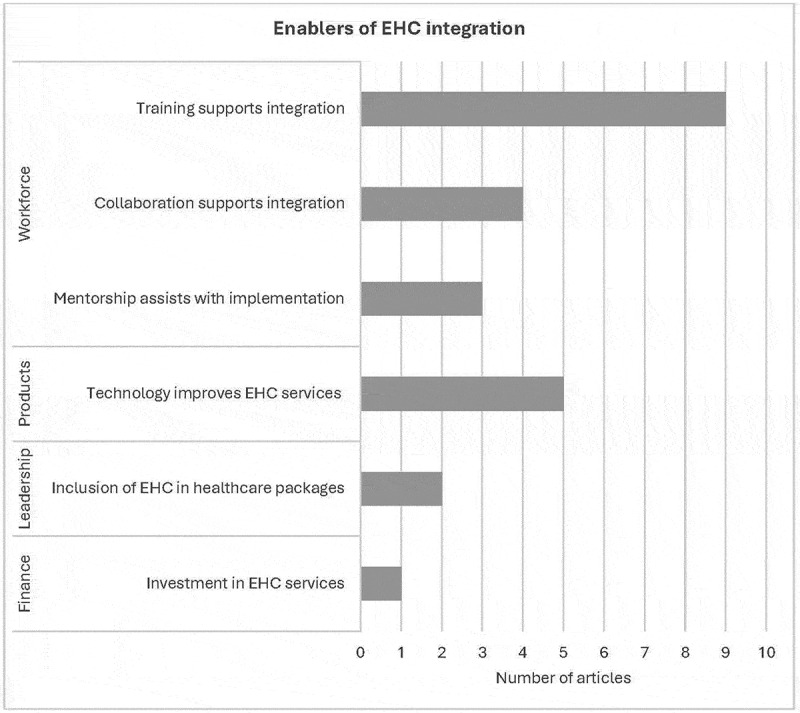
Distribution of enablers mapped to the WHO framework [1].

We describe the key themes identified from the included papers that enabled EHC integration, linking them to the health system-building blocks, whilst also considering the level of integration using the EHC service delivery framework [1]. The dominant strategies enabling EHC integration were training, collaboration, mentorship, technology, inclusion of EHC in health-care packages and investment (Figure 3).

#### Workforce

##### Training supports integration

The continuous shortage of skilled HCWs is a major challenge in many LMICs. One of the approaches to address this shortage is training CHWs to conduct specialised services such as hearing screening [40,42,49]. The micro-level integration strategy of task shifting or sharing is, thus, enabled by offering CHWs specialised training to conduct these services, also affecting the quality and coverage of services [41].

Several papers offered experiences on how to conduct or strengthen training of CHWs using in-person, online or blended approaches [39,41–43,49]. Four micro-level studies involved focussed training programmes aimed at achieving desired health outcomes, such as improved access to hearing screening aligned to the needs of communities [39,41,42,47]. CHWs in India enabled integration through training using a targeted 6-week programme to expand EHC services which covered fitting hearing aids and providing counselling [39]. A Ugandan study approached training using hybrid methods which consisted of a short 2-day in-person programme followed by remote supervision of CHWs using technology, such as WhatsApp [41]. Only one study in Malawi used the standardised WHO Basic and Intermediate training modules on Primary Ear and Hearing Care to train CHWs in basic EHC services which aim to relieve the service delivery burden on secondary and tertiary levels [42]. In Ghana, the implementation of community-based hearing programmes conducted under the supervision of specialised HCWs showed to be useful [47]. A study in Cambodia described how non-governmental organisations and public hospitals have addressed the gap in specialised training for HCWs to enable task sharing and expand services which includes hearing screening and treatment [50]. These papers described various approaches to training CHWs which resulted in addressing workforce challenges and expanding EHC service delivery particularly in underserved communities.

##### Mentorship assists integration

Training can address EHC staff shortages; however, retaining skilled staff in rural settings is another challenge facing the health system. Mentorship of staff can be seen as a system software enabler of integration, going beyond training, which supports staff retention, and enables skilled staff to implement services effectively. For instance, in Uganda, CHWs were trained and further supported remotely, thereby providing continuous support for trained staff at micro-level and effective implementation [41]. Another study showed that micro-level approaches which includes continuous supervision and guidance by specialised HCWs beyond training programmes support retention and competency of trainees [40]. Furthermore, adapting training to include mentorship and supervision improved staff morale as HCWs were more motivated and satisfied [40]. Similarly, Eubank et al. [49] argued that training programmes can benefit from including feedback mechanisms in community-based assessment programmes.

##### Collaboration supports integration

Collaborative efforts between stakeholders, particularly communities and professionals, act as system software enablers of EHC integration through creating formal and informal partnerships. For instance, Indian partnerships at micro- and meso-level between HCWs and staff at schools facilitated the successful implementation of remote hearing screening programmes and parents’ acceptance of EHC services [43]. Two studies highlighted that professional collaboration between specialised HCWs such as audiologists or ear, nose and throat (ENT) specialists and local HCWs (e.g. CHWs) at both micro- and meso-level was crucial to ensure integration of services at community level [41,48]. In India, the implementation of remote audiological services at meso-level through the collaborative efforts of CHWs and specialised HCWs focussed on increasing access to hearing screening in communities, resulting in improved quality of care, accessibility and better screening outcomes [49].

#### Products

##### Technology improves EHC services

Advances in technology have shifted the requirement for specialised equipment and reduced the need for specialised screening facilities, improving EHC service delivery in low-resource settings [43]. The use of technology at micro-level in India has impacted quality of care by improving capturing of health information, triaging of patients and the referral process [43]. Micro-level approaches in South Africa addressed the lack of infrastructure using low-cost alternatives to replace expensive hearing screening devices and showed that minimal training is required to conduct hearing screening using mobile technology [44]. Most technologies require some form of internet connectivity but equipment which does not require internet for data capturing can be considered in resource-limited settings, as demonstrated in Peru [48]. Most CHWs perceived mobile applications as user-friendly and efficient, as described in India [43,44]. Implementation of EHC services using technology at community-level reduced travel time to clinics, use a familiar environment and was positively perceived by patients and caregivers [43].

A particular approach noted in Ghana was telemedicine initiatives, such as audiological hubs [47]. These hubs can serve as collaborative centres to improve service delivery and referral systems by linking surrounding healthcare facilities and healthcare professionals [47,51]. Thus, establishing a centre of excellence for EHC to support service delivery and quality of care.

#### Leadership

Leadership and governance are essential to ensure that health systems develop equitable access to all services, through combining effective oversight, coalition-building and regulation, while considering implementation and accountability [1].

##### Inclusion of EHC in health-care package

Given that leadership and governance include setting overall policy frameworks for healthcare and the WHO encourages the inclusion of EHC services at primary level, the inclusion of NBHS in primary care in Ghana and Peru appears to reflect positive leadership and governance [1]. In the Peruvian context, the importance of NBHS programmes for equitable access to EHC was highlighted through a meso-level integration approach assisting with organised service delivery through interprofessional integration [48]. In Ghana, the effectiveness of including NBHS in primary care packages at micro-level was shown by using alternative service delivery methods, such as telemedicine to address coverage and equitable access [47].

#### Finance

##### Investment in EHC services

Only one micro-level study highlighted that prioritising EHC services through investment could enable integration of EHC into existing primary health and community-based services supported by an efficient referral pathway [40]. The study noted that sustainability initiatives, such as monetary incentives, can be used to improve staff retention in rural areas [40].

### Barriers to integration of ear and hearing health-care services

**Figure 4.**
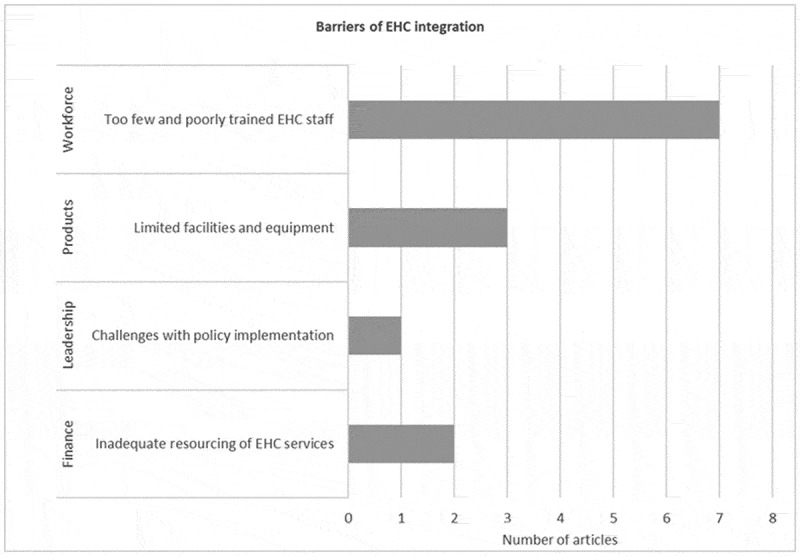
Distribution of barriers mapped to the WHO framework [1].

Although the WHO EHC framework focuses on enablers of integration, the framework also proved useful in identifying potential barriers to integration, factors which hindered integration approaches. The similarity of identified barriers to integration across included papers suggests that other LMICs might face similar challenges (Figure 4). Shortage of workforce was the most prominent barrier followed by limited facilities and equipment, high cost of EHC services and challenges with policy implementation.

#### Workforce

##### Too few and poorly trained EHC staff

HCWs are the cornerstone of any health system. Several studies highlighted human resource challenges that included shortages of skilled staff and insufficient training of HCWs [39,40,42,43,51,52]. For instance, micro-level integration was only possible at the Korle-Bu Teaching Hospital located in the Greater Accra Region, which offers EHC services for infants, due to the general lack of skilled HCWs in Ghana [47]. In Malawi, micro-level integration showed that HCWs often allocate time to other services, rather than EHC [42].

Although training of CHWs can assist with addressing HCW shortages, this approach has its limitations [51]. O’Donovan et al. [39] described challenges due to the variability in training approaches in LMICs. In the Indian setting, micro-level integration approaches highlighted the practical and logistical challenges with training CHWs in telemedicine to conduct community-based hearing screening [43]. The COVID-19 pandemic led to a rapid shift to telemedicine globally, which posed implementation challenges for HCWs, such as adequate training and adapting, to telemedicine [52]. Furthermore, issues around the quality of training included access to resources, training materials and trainers for CHW training programmes as well as appropriate assessments to determine the competency and effectiveness of training programmes [39–41]. The scope of training modules may also not be adequate. For example, audiologists in Malaysia felt that their training was inadequate as they were not prepared to counsel and manage patients with hearing loss [40]. Consequently, the lack of supporting services such as counselling was highlighted by caregivers of hearing-impaired children as a contributing factor for the rejection of hearing devices and low acceptance [40].

#### Products

##### Limited facilities and equipment

Factors such as lack of resources can influence a country’s ability to implement integration approaches. In South Africa, for example, although there is an Integrated School Health Policy which requires that each student is screened for hearing when entering school, this is not being implemented due to lack of equipment and infrastructure, as well as too few trained personnel [44]. India has a similar approach to hearing screening at schools, but this system has also experienced setbacks to implementation for instance connectivity issues in remote areas for telemedicine and logistics (e.g. space, staffing) with conducting hearing screening at schools [43]. One study in Malawi described that HCWs identified a lack of equipment for proper diagnosis, limited medication and supporting structures for staff as major constraints in providing much-needed EHC services [42].

#### Leadership

Lack of leadership generates policy challenges for the implementation of integration.

##### Challenges with policy implementation

Only one study explicitly identified as a challenge, the lack of policy relating to universal NBHS [53]. In South Africa, the lack of mandatory neonatal and infant hearing screening programmes, due to priority being given to infectious diseases, results in late diagnosis and delayed interventions [53]. Nonetheless, the lack of health system leadership was a commonly identified integration challenge [40,44,47,48,51,53].

#### Finance

##### Inadequate resourcing of EHC services

Only two studies addressed this issue. The lack of continued funding for community-based EHC services at micro-level in India was judged as negatively influencing hearing screening, interventions and follow-up rates [43]. A study, from South Africa, reported an evaluation of low-cost options for hearing screening, using technology or mobile screening devices to improve access and integration of services [44]. However, these cost-effective hearing screening techniques require continued funding from governments to be effective.

### Healthcare seeking behaviour: factors impacting the use of integrated EHC services

Looking beyond health system factors, we include the theme of ‘healthcare seeking’ in our findings. It was identified, through inductive analysis of the literature reviewed, as a critical factor influencing the reported experiences of integration. Although not part of the WHO framework it adds value in understanding the factors influencing the utilisation of integrated services, shedding further light both on the health system and additional issues which need to be considered with integration approaches. A range of factors were identified as positively or negatively influencing patients’ decisions to use existing or newly integrated EHC services.

#### Access and ability to pay

The geographical location of healthcare facilities can be a major stumbling block for integration as many specialised facilities are located far from rural communities with challenging terrains and limited transport options [40,42]. Micro-level studies in South Africa investigate the challenges with NBHS implementation and follow-up rates identifying lack of transport, funds for transport and distance to the facility as key barriers [40,54]. Two other South African studies described challenges with transport costs, unreliable public transport and distances to healthcare facilities as a major obstacle to access EHC services [55,56]. For low-income households’ additional expenses, such as out-of-pocket (OOP) payments for medical costs, can deter patients from actively seeking healthcare. In Nigeria, inaccessibility of EHC services was described by caregivers of hearing-impaired children due to the high cost of hospital care [51]. A micro-level study in South India showed that although more than 80% of mothers were willing to pay for children to be screened for hearing, only 24% could afford to pay for EHC services [39]. In Cambodia, it was highlighted that the high cost of hearing devices and cochlear implants is not affordable for most of the population leaving patients without viable treatment options [50]. Some studies offered ways to reduce OOP costs for instance, in India a micro-level study showed that telemedicine is a useful tool to enable EHC integration by improving access to services in rural communities [43]. Healthcare providers bring the services closer to the communities through initiatives like telemedicine to reduce costs such as transport, screening time and loss of income and improve acceptability of services [45].

#### Using referral systems

The effective management of patients and referrals are important aspects of a robust health system and were highlighted as an influencing factor in the use of EHC services. Community-based hearing screening programmes in Nigeria and Uganda, which included CHWs in referral pathways showed that their inclusion at micro level assisted with effective referral for specialised services and reduced inappropriate referrals [41,51].

In Malawi, stakeholders identified the absence of acute symptoms, limited availability of EHC services, transport challenges, such as distance to hospitals and lack of transport, lack of information, long waiting periods, high OOP, lack of awareness and communication as reasons for patients not attending follow-up visits [42]. This study recommended that referral pathways should consider context-specific issues, such as geographical and financial challenges, and include multi-dimensional teams (e.g. CHWs, specialised HCWs and health centre managers) [42]. The experience in Nigeria showed that although referral pathways were in place, this did not lead to improved follow-up visits as prevailing issues such as the cost of hospital care prevented utilisation by patients and caregivers [51]. In Cambodia, ineffective referral pathways and low utilisation of EHC services were impacted by competitive processes by health-care facilities to retain income or their reputation, lack of resources, lack of awareness and prioritisation [50]. This study highlighted the need for a more robust referral pathway linking various levels of healthcare. Finally, a study in Bangladesh among young children revealed that most caregivers do not attend referrals due to the absence of acute symptoms, even though they were encouraged by CHWs [40].

#### Communication and awareness

Another theme identified was a lack of awareness of EHC services and knowledge of hearing loss among communities. In a Nigerian study, technology was used to educate communities with the aim of raising awareness and increasing knowledge of hearing screening interventions to increased utilisation of services [51]. A micro-level South African study showed that exposing caregivers and patients to persons affected by hearing loss can act as an enabler as this can help with understanding the consequences of not attending follow-up appointments [45]. Similarly, a study conducted with mothers in South Africa described limited knowledge of infant hearing loss risk-factors and low awareness of EHC services [53]. Communication and awareness need to consider culture and its influence on healthcare seeking behaviour.

Communities accepted EHC interventions more easily when they were conducted by organisations with a good track record in communities. For example, a non-profit organisation (NPO) successfully implemented hearing screening at meso-level in rural communities in Peru based on trust and existing partnerships [48]. The attitude and communication skills of audiologists at micro-level in South Africa had a positive effect on follow-up rates and utilisation of services [45]. Community-based EHC services delivered by local HCWs were positively received by patients in South Africa and helped to build trust in health-care system [55]. Furthermore, micro-level studies in LMICs showed that community participation is important to raise awareness and can facilitate acceptance and strategic planning of healthcare initiatives [39].

## Discussion

This systematic review summarised the key integration approaches for ear and hearing care services reported from LMIC health systems. The pool of eligible studies from LMICs was very low (17 papers) emphasising the gap in knowledge and likely lack of interest or priority in EHC services in developing countries. This is concerning given the significant burden of disease of hearing loss in LMICs. Similar findings were revealed in a recent review by Waterworth et al. [40].

**Figure 5.**
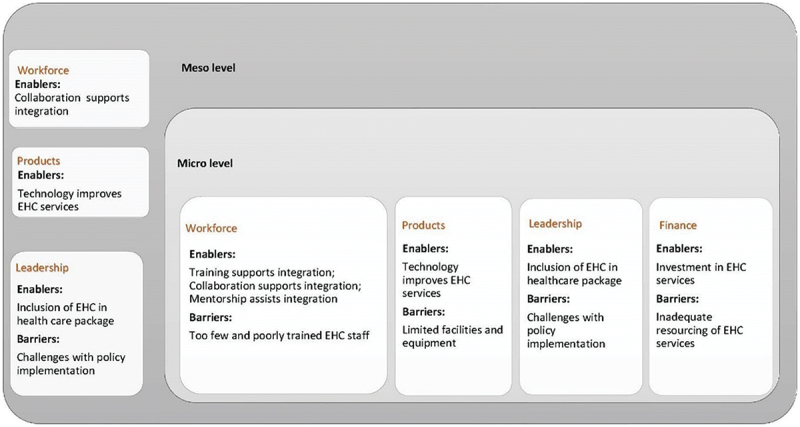
Key themes categorised according to the level of integration (source: Author).

The review investigated EHC integration approaches by adapting the Valentijn framework [16] to categorise the dimensions and level of integration, as well as using the WHO EHC framework [1] to identify enablers and barriers to EHC integration. The use of these frameworks supported systematic synthesis of the available qualitative evidence and understanding of EHC integration approaches through a health systems lens, as summarised in Figure 5. However, during data synthesis, we found it useful to add consideration of health-care seeking behaviour to allow for the full range of identified factors influencing experiences of EHC integration.

### Integration approaches for EHC service delivery

Most of the literature reviewed described integration at micro or individual level with a few studies at meso or organisational level and no studies at macro or systems level. Similar trends have been observed in broader literature, which reviewed integrated care in LMICs and HICs [57]. The lack of macro-level integration might be due to the limited number of LMICs with EHC health policies, perhaps also reflecting a lack of leadership around EHC services. Studies mainly approached integration at service and clinical levels followed by professional, normative and organisational levels, often focussed on addressing accessibility constraints in rural areas through service delivery models.

EHC integration approaches in LMICs addressed the critical shortage of HCWs by focussing on clinical and service-level integration [39–42]. These approaches incorporated CHWs into the EHC workforce through task shifting/sharing, by training CHWs with the goal to expand services and improve referral mechanisms. Several studies described service-level integration through targeted programmes that aims to address accessibility to EHC services [39,43–47]. A combination of integration approaches was used to support integration with both horizontal and vertical approaches shown to be useful for EHC integration. Previous studies also showed that combining horizontal and vertical integration can be beneficial for approaches working across and within building blocks and across various health system functions [17]. However, broader literature shows that integration approaches in HICs focus more on improving system processes rather than individual services [57]. Given the lack of such approaches in LMICs, there is a need to shift integration strategies to the macro-level, such as national governments, with the goal of improving existing structures and creating new collaborative efforts. As governments are not prioritising policies for universal hearing screening and policy implementation in LMICs, this is resulting in late diagnosis and delayed treatment [48,53]. For instance, policy initiatives, such as the inclusion of NBHS programmes to detect and treat hearing loss at an early age, is recommended by the WHO [1] but has not been achieved in most LMICs, in contrast to high-income countries [58]. Another contrast between countries is that, as observed in this review, the aim of integration in most LMICs is to increase service delivery, whereas in HICs the focus is more on changing patterns of use towards more cost-effective services [57].

### Enabling and constraining EHC integration

This review revealed that success in addressing the critical shortage of EHC healthcare workers is a key factor enabling or constraining integration in LMICs. To enable integration, factors, such as training, collaboration and mentorship, supported integration by increasing the number of healthcare workers supporting EHC service delivery [39–43,47,49]. Task shifting or sharing is recommended by the WHO as a tool to support EHC integration [1] and, as shown in this review, requires targeted training programmes to upskill CHWs with the goal of delivering EHC services at community level [59,60]. Task shifting or sharing approaches to address HCW shortages have also been shown to be useful in LMICs for hearing screening and other health conditions [17,59–61]. However, this review showed the continued need to standardise training of CHWs in EHC service delivery to ensure a continuum of quality healthcare and to offer continued support through mentorship and supervision beyond short-lived training programmes [40,41,59,62]. Addressing the software components of the health system, such as mentorship, motivation and acceptance of training interventions, can further assist with staff retention and positively impact service integration. The review suggests that integration approaches should consider a more collaborative, community centred approach and be adaptable [40,42,51,53,54] also highlighted by previous studies [59,61]. Despite the efforts made towards EHC integration, there is still a lack of investment and sustainability plans for EHC services [39,40,43,50,52]. The WHO’s push towards integrating EHC services into national health systems is a key aspect for addressing these health inequities [1,60].

### Healthcare seeking behaviour and EHC integration

Another key feature of this review was the inclusion of healthcare seeking as a systemic issue influencing the use of integrated or newly integrated EHC services. Healthcare seeking behaviour is influenced by accessibility of EHC services (considering overall availability, service location and affordability). The availability of EHC services, especially specialised services, such as cochlear implants, simply does not meet the needs in LMICs [60,63,64]. We also identified that the geographical location of specialised healthcare facilities plays an important role in referral attendance and follow-ups [40,42]. This review further highlighted the importance of referral systems to incorporate CHWs and foster collaboration between HCWs throughout different levels of the health system [41,42,49,51].

The available literature does, however, suggest that empowering communities and focussing on knowledge gaps related to hearing loss and EHC services can encourage EHC service use [45,51,53]. Through engaging with communities, the implementation strategies can be adapted to local context to be more culturally sensitive and acceptable to patients and caregivers [1,39,45,48].

#### Limitations

Although a comprehensive search approach was used, a limitation of this study was that the focus was only on LMICs. It would be useful to broaden the scope of the review to include HICs as some areas in these countries are also under-resourced and might face issues not described in this study. It would also be interesting to compare EHC integration approaches in LMICs and HICs. Only focusing on English language literature might exclude relevant experiences written in other languages. Finally, although this review did not include a quality assessment processes, it only included peer-reviewed articles which would have already undergone a quality control process.

## Conclusion

This study has described how EHC services are integrated in LMICs, as well as the factors, enabling or constraining integration, through a health systems lens. It also highlighted the influence of healthcare seeking behaviour on integration approaches. This comprehensive overview and lessons learnt were drawn from experiences of EHC integration in LMICs and may be useful to policy makers and those involved in implementing integration of EHC services into the wider healthcare system. Further, despite the limited published literature on EHC integration in LMICs, there might be other approaches to integration or experiences that were not considered in this study. This reiterates the need for future research on EHC service integration in low-resource settings to help address the ongoing need for these services.

## Supplementary Material

PRISMA_2020_checklist_Integration of EHC services.docx

Supplementary 1.docx
